# Hypertension and hyperglycaemia are positively correlated with local invasion of early cervical cancer

**DOI:** 10.3389/fendo.2023.1280060

**Published:** 2023-12-12

**Authors:** Tiantian Shen, Jing Zhao, Wenhan Li, Xiaoman Wang, Yumei Gao, Zehua Wang, Sha Hu, Jing Cai

**Affiliations:** Department of Obstetrics and Gynecology, Union Hospital, Tongji Medical College, Huazhong University of Science and Technology, Wuhan, China

**Keywords:** metabolic disorders, pathological characteristics, cervical cancer, hypertension, obesity, hyperglycemia

## Abstract

**Background:**

Metabolic disorders are involved in the development of numerous cancers, but their association with the progression of cervical cancer is unclear. This study aims to investigate the association between metabolic disorders and the pathological risk factors and survival in patients with early cervical cancer.

**Methods:**

Patients with FIGO IB1 (2009) primary cervical cancer who underwent radical hysterectomy and systematic pelvic lymph node dissection at our institution from October 2014 to December 2017 were included retrospectively. Clinical data regarding the metabolic syndrome and surgical pathology of the patient were collected. The correlations between metabolic disorders (hypertension, hyperglycemia, and obesity) and clinicopathological characteristics as well as survival after surgery were analyzed.

**Results:**

The study included 246 patients with clinical IB1 cervical cancer, 111 (45.1%) of whom had at least one of the comorbidities of hypertension, obesity, or hyperglycemia. Hypertension was positively correlated with parametrial invasion and poorly differentiated histology; hyperglycemia was positively correlated with stromal invasion; obesity was negatively associated with lymph node metastasis; but arbitrary disorder did not show any correlation with pathologic features. Hypertension was an independent risk factor for parametrial invasion (OR=6.54, 95% CI: 1.60-26.69); hyperglycemia was an independent risk factor for stromal invasion (OR=2.05, 95% CI: 1.07-3.95); and obesity was an independent protective factor for lymph node metastasis (OR=0.07, 95% CI: 0.01-0.60). Moreover, the patients with hypertension had a significantly lower 5-year OS rate (70.0% vs. 95.3%, *P*<0.0001) and a significantly lower 5-year PFS rate than those without hypertension (70.0% vs. 91.2%, *P*=0.010).

**Conclusion:**

Hypertension and hyperglycemia are positively associated with local invasion of early cervical cancer, which need to be verified in multi-center, large scale studies.

## 1 Introduction

Noncommunicable diseases are the leading cause of mortality on the global scale (1). Metabolic syndrome is the most prevalent condition among these noncommunicable disorders. According to global statistics, more than one-third of adults in the United States suffer from metabolic syndrome, of which more than 50% are older than 60, and in some Asian countries, the prevalence is as high as 10% (2), making it a pervasive and potentially fatal condition worldwide. Metabolic syndrome is characterized by obesity, insulin resistance, hypertension, and hyperlipidemia (1) and is associated with the progression of diseases such as type 2 diabetes mellitus, cardiovascular disease, and nonalcoholic steatohepatitis (3, 4). Recent epidemiological studies have indicated that metabolic syndrome is also closely associated with the development of malignant tumors such as liver, bladder, renal, endometrial, pancreatic, and breast cancers (5–11). Moreover, recovery from metabolic syndrome by making lifestyle adjustments is associated with a reduced risk of pancreatic cancer compared to persistent metabolic syndrome, implying that alterations in metabolic syndrome can affect pancreatic cancer risk (12). Experiments have also shown that organismal metabolism can influence the development and progression of cancers, for instance, by modulating diet to control tumor progression (13–15). The introduction of a high-fat diet in a mouse model has been observed to increase the growth and metastasis of a primary tumor (16). Hyperglycemia promotes upregulation of mitochondrial protein GRP75 in megakaryocytes, which increases platelet activation to promote cancer metastasis in the mouse model of melanoma (17). However, how alterations in metabolic levels affect cancer risk and progression is not yet fully understood (18).

Cervical cancer ranks as the fourth most prevalent malignant tumor in females worldwide in terms of incidence and mortality (19), representing a substantial global health challenge. A retrospective study suggested that metabolic syndrome increases the risk of cervical epithelial cell abnormalities and persistent HPV infection (20–22). Large prospective epidemiologic studies and retrospective analyses have found that metabolic abnormalities may increase the risk of cervical cancer (23, 24). Nevertheless, the correlation between metabolic syndrome and cervical cancer progression is currently uncertain. In terms of mechanisms, there is limited relevant research. Hyperglycemia might accelerate the growth of cervical cancer by increasing the expression of the proteasome alpha 2 subunit (25). In obese women, the communication between tumor cells and adipocyte-derived stem cells may be enhanced, which can regulate the expression of diverse chemokines that favor malignancy-associated capacities such as migration (26). So, we were prompted to explore the clinical evidence for the role of metabolic abnormalities in cervical cancer progression, which can fuel studies on underlying mechanisms that may help to develop novel strategies for treatment of cervical cancer. Moreover, clarifying the risk factors of tumor progression can be important to guide clinicians towards better therapeutic choices, such as radiochemotherapy for more advanced disease (27).

In early-stage cervical cancers, local invasion is the principal way of tumor to spread, and lymphatic metastatic can be found postoperatively in about 15% of clinically early tumors (28, 29). Therefore, we designed a retrospective study to preliminarily investigate the association between metabolic disorders and pathological features of cervical cancer, including tumor size, parametrial invasion, stromal invasion, and lymph node metastasis. To minimize the effect of confounding factors, only patients undergoing radical surgery at clinical stage IB1 (FIGO, 2009) were included in this study. This is the first study to examine the relationship between metabolic disturbance and the pathological characteristics of cervical cancer, and it is anticipated that it will provide clinical evidence for the body’s metabolic role in the progression of cervical cancer.

## 2 Methods

### 2.1 Study population

This study involved a cohort of patients diagnosed with clinical stage IB1 cervical cancer who underwent surgery at our institution between October 2014 and December 2017. The studies were conducted in accordance with the local legislation and institutional requirements. The study protocol was approved by the ethics committee of Tongji Medical College of Huazhong University of Science and Technology. Patients met the following inclusion criteria: (1) surgical pathology diagnosis of primary cervical cancer with any type of pathology; (2) preoperative stage IB1 diagnosis according to 2009 FIGO; and (3) radical C-type surgery and postoperative pathology testing at our institution. Patients who met the following criteria were excluded: (1) received preoperative neoadjuvant chemotherapy or radiotherapy; (2) had incomplete data regarding blood glucose, blood pressure, body mass index (BMI), tumor size, or important pathological details (such as pathological type, histologic grading, parametrial invasion, stromal invasion, lymphovascular space involvement (LVSI), removed lymph nodes, and lymph node metastasis); and (3) had a combination of other malignant tumors.

### 2.2 Data collection and diagnostic criteria

Data regarding the patient’s age, preoperative BMI, blood pressure, glucose levels, and medical history related to metabolism (such as hypertension, abnormal glucose tolerance, and obesity) were obtained from the patient’s medical records. The tumor size was determined based on preoperative imaging and postoperative pathology data, with the largest lesion being selected for analysis. Additional information collected from medical records included clinical FIGO staging, pathological type, histology grading, parametrial invasion, stromal invasion, LVSI, removed lymph nodes, and lymph node metastasis. At our hospital, during the period of October 2014 and December 2017, clinical staging of cervical cancer was mainly based on physical examination and imaging of pelvic organs. The most frequently used imaging examination was magnetic resonance imaging, while computed tomography and ultrasound were used as alternatives occasionally. In this investigation, we applied the diagnostic criteria for obesity, hypertension, and hyperglycemia in metabolic syndrome established by the Chinese Diabetes Association in 2004 (30). (1) obesity: ≥25.0 kg/m^2^; (2) hyperglycemia: fasting blood glucose ≥6.1 mmol/L; postprandial blood glucose ≥7.8 mmol/L; or diabetes mellitus diagnosed and treated; (3) blood pressure ≥140/90 mmHg (1 mmHg = 0.133333 kPa) or hypertension diagnosed and treated. The exclusion of lipids from the study was due to their not being routinely tested preoperatively in our hospital, hence posing challenges in the collection of data pertaining to lipid metabolism. The survival outcomes were collected from the medical records in combination with telephone follow-up data. Overall survival (OS) was defined as the interval between the date of surgery and the date of death or to the end of follow-up. Progression-free survival (PFS) was defined as the interval between the date of surgery and the diagnosis of recurrence or to the end of follow-up.

### 2.3 Statistical analysis

SPSS 27.0 software was applied to process the data. Survival curves were generated using GraphPad Prism 10.0. Continuous variables that followed a normal distribution are presented as the mean ± standard deviation, while nonnormally distributed continuous variables are presented as the median (range). The t test and the Mann−Whitney U test were used to compare normally distributed and nonnormally distributed variables, respectively. Categorical variables were compared using the chi-square (χ2) test—the ordinary χ2 test for n ≥ 40 and T ≥ 5 in chi-square tests. When n ≥40 and 1≤T<5, continuity correction was implemented. Fisher’s test was applied when n<40 or there was T<1. Univariate and multivariate logistic regression analyses were used to assess the risk of tumor parametrial invasion, stromal invasion, and lymph node metastasis. Additionally, those variables with *P* values <0.05 in univariate logistic regression analyses were included in multivariate logistic regression analysis. The results were expressed as odds ratios (ORs) and 95% confidence intervals (95% CIs). Survival curves were plotted by the Kaplan-Meier method and compared using the log-rank test. Two-sided *P* values were considered statistically significant at *P* < 0.05.

## 3 Results

### 3.1 Baseline characteristics of patients

This investigation enrolled a total of 246 patients with clinical stage IB1 cervical cancer, and the median age of the patients was 47 (range 25-75) years. A total of 111 patients (45.1%) had at least one of the metabolic disorders of hypertension, hyperglycemia or obesity, with hypertension accounting for 28 patients (11.4%), hyperglycemia accounting for 83 patients (33.7%) and obesity accounting for 47 patients (19.1%). There were 170 cases (69.1%) with a tumor size of 2-4 cm, with squamous carcinoma accounting for 76.8% (189/246). Patients with parametrial invasion, stromal invasion (outer 1/3), LVSI, and lymph node metastases were found in 21 (8.5%), 68 (27.6%), 80 (32.5%), and 32 (13.0%) patients, respectively (**Table 1**).

**Table 1:** Baseline characteristics for 246 clinical stage IB1 cervical cancer patients.

Characteristics	Number of patients (%)
Age (years)	
Median, range	47, 25-75
<50	159 (64.6)
≥50	87 (35.4)
Menopausal	
Yes	163 (66.3)
No	81 (32.9)
Unknown	2 (0.8)
Hyperglycemia	
Negative	163 (66.3)
Positive	83 (33.7)
Obesity	
BMI median, range	22.3, 16.7-35.6
Negative	199 (80.9)
Positive	47 (19.1)
Hypertension	
Negative	218 (88.6)
Positive	28 (11.4)
Metabolic disorder^*^	
0	135 (54.9)
1	72 (29.3)
2	30 (12.2)
3	9 (3.6)
Tumor Size	
< 2 cm	76 (30.9)
2-4 cm	170 (69.1)
Pathologic type	
Squamous cell cancer	189 (76.8)
Adenocarcinoma	41 (16.7)
Adenosquamous cancer	8 (3.3)
Neuroendocrine cancer	6 (2.4)
Others	2 (0.8)
Histologic grading	
G1	27 (11.0)
G2	148 (60.1)
G3	71 (28.9)
Parametrial invasion	
Negative	225 (91.5)
Positive	21 (8.5)
Stomal invasion	
Negative	178 (72.4)
Positive	68 (27.6)
LVSI	
Negative	166 (67.5)
Positive	80 (32.5)
Lymph node metastasis	
Negative	214 (87.0)
Positive	32 (13.0)

*Hyperglycemia, obesity and hypertension; the numbers (0, 1, 2, and 3) represent the variety of comorbidities. BMI, body mass index; G1, well differentiated tumor; G2, intermedium differentiated tumor; G3, poorly differentiated tumor; LVSI, lymphovascular space involvement.

### 3.2 Correlation of metabolic abnormalities with clinicopathologic characteristics

Patients older than 50 were more likely to have hypertension and obesity, whereas hyperglycemia was not significantly associated with patient age. Moreover, the proportion of positive parametrial invasion patients with comorbid hypertension (28.6%) was significantly higher than the proportion of negative parametrial invasion patients with comorbid hypertension (9.8%, *P*=0.025); the proportion of positive stromal invasion patients with comorbid hyperglycemia (44.1%) was significantly higher than the proportion of negative stromal invasion patients with comorbid hyperglycemia (29.8%, *P*=0.033). In addition, the proportion of obese patients with lymph node metastasis was significantly lower than the proportion of obese patients without lymph node metastasis (3.1% vs. 21.5%, *P*=0.014). There was no statistical correlation between arbitrary metabolic abnormalities and pathologic risk factors in patients (**Table 2**).

**Table 2:** Correlation of metabolic disorders with clinicopathologic characteristics in patients with IB1 cervical cancers (N=246).

Variable	Patients	Hypertension		Hyperglycemia		Obesity		Arbitrary metabolic disorder *	
		N (%)	*P* value	N (%)	*P* value	N (%)	*P* value	N (%)	*P* value
Age (years)			<0.001		0.190		0.004		0.019
<50	159	9 (5.7)		49 (30.8)		22 (13.8)		63 (39.6)	
≥50	87	19 (21.8)		34 (39.1)		25 (28.7)		48 (55.2)	
Menopausal			<0.001		0.040		0.005		0.007
Yes	81	18 (22.2)		34 (42.0)		23 (28.4)		46 (56.8)	
No	163	10 (6.1)		47 (28.8)		22 (13.5)		63 (38.7)	
Tumor size			0.473		0.632		0.855		0.935
<2 cm	76	7 (9.2)		24 (31.6)		14 (18.4)		34 (44.7)	
2-4 cm	170	21 (12.4)		59 (34.7)		33 (19.4)		77 (45.3)	
Histologic type			0.807		0.176		0.467		0.409
Squamous cell cancer	189	21 (11.1)		68 (36.0)		38 (20.1)		88 (46.6)	
Others	57	7 (12.3)		15 (26.3)		9 (15.8)		23 (40.4)	
Histologic grading			0.009		0.229		0.112		0.092
G1-G2	175	14 (8.0)		55 (31.4)		29 (16.6)		73 (41.7)	
G3	71	14 (19.7)		28 (39.4)		18 (25.4)		38 (53.5)	
Parametrial invasion			0.025		0.600		0.149		0.485
Negative	225	22 (9.8)		77 (34.2)		40 (17.8)		100 (44.4)	
Positive	21	6 (28.6)		6 (28.6)		7 (33.3)		11 (52.4)	
Stromal invasion			0.907		0.033		0.715		0.070
Negative	178	20 (11.2)		53 (29.8)		33 (18.5)		74 (41.6)	
Positive	68	8 (11.8)		30 (44.1)		14 (20.6)		37 (54.4)	
LVSI			0.636		0.998		0.657		0.764
Negative	166	20 (12.0)		56 (33.7)		33 (19.9)		76 (45.8)	
Positive	80	8 (10.0)		27 (33.8)		14 (17.5)		35 (43.8)	
Lymph node metastasis			0.609		0.749		0.014		0.584
Negative	214	23 (10.7)		73 (34.1)		46 (21.5)		98 (45.8)	
Positive	32	5 (15.6)		10 (31.3)		1 (3.1)		13 (40.6)	

* Hypertension, hyperglycemia, or obesity.

P value, χ2 test.

G1, well differentiated tumor; G2, intermedium differentiated tumor; G3, poorly differentiated tumor; LVSI, lymphovascular space involvement.

### 3.3 Univariate analysis of pathologic risk factors

Based on the results of the above correlation analysis, we further investigated the risk factors for parametrial invasion, stromal invasion, and lymph node metastasis (see **Table 3**). Univariate logistic regression results revealed that hypertension (OR=3.69, 95% CI: 1.30-10.48), tumor size, LVSI, stromal invasion and lymph node metastasis were all risk factors for parametrial invasion. Hyperglycemia (OR=1.86, 95% CI: 1.05-3.31), menopause, tumor size, LVSI, parametrial invasion and lymph node metastasis were associated with an elevated risk of stromal invasion. Tumor size, LVSI, parametrial invasion and stromal invasion increased the risk of lymph node metastasis, while obese patients had a lower risk of lymph node metastasis (OR=0.12, 95% CI: 0.02-0.89).

**Table 3:** Univariate regression analysis of risk factors for parametrial invasion, stromal invasion, and lymph node metastasis.

Variable	Parametrial invasion			Stromal invasion			Lymph node metastasis		
	OR	95% CI	*P* value	OR	95% CI	*P* value	OR	95% CI	*P* value
Age ≥50 years (yes vs. no)	1.41	0.57-3.50	0.455	1.68	0.95-2.97	0.077	0.47	0.19-1.13	0.093
Menopause (yes vs. no)	1.57	0.63-3.90	0.329	1.94	1.09-3.45	0.025	0.52	0.22-1.26	0.150
Tumor size ≥ 2cm (yes vs. no)	4.66	1.06-20.52	0.042	3.96	1.84-8.50	<0.001	5.01	1.48-16.98	0.010
Pathologic type of SCC (yes vs. no)	1.31	0.42-4.06	0.640	1.39	0.69-2.78	0.353	3.26	0.96-11.14	0.059
Histologic grading (G3 vs. G1-G2)	1.97	0.79-4.91	0.145	1.04	0.56-1.92	0.906	0.96	0.42-2.19	0.921
LVSI (yes vs. no)	10.93	3.54-33.74	<0.001	3.75	2.08-6.75	<0.001	3.15	1.48-6.73	0.003
Parametrial invasion (yes vs. no)	NA	NA	NA	10.65	3.72-30.45	<0.001	6.59	2.51-17.31	<0.001
Stromal invasion (yes vs. no)	10.65	3.72-30.45	<0.001	NA	NA	NA	2.31	1.07-4.95	0.032
Lymph node metastasis (yes vs. no)	6.59	2.51-17.31	<0.001	2.31	1.07-4.95	0.032	NA	NA	NA
Hypertension (yes vs. no)	3.69	1.30-10.48	0.014	1.04	0.56-1.92	0.906	1.54	0.54-4.39	0.421
Hyperglycemia (yes vs. no)	0.77	0.29-2.06	0.601	1.86	1.05-3.31	0.035	0.88	0.40-1.95	0.750
Obesity (yes vs. no)	2.31	0.88-6.10	0.09	1.14	0.57-2.29	0.715	0.12	0.02-0.89	0.038

SCC, squamous cell cancer; G1, well differentiated tumor; G2, intermedium differentiated tumor; G3, poorly differentiated tumor; LVSI, lymphovascular space involvement; OR, odds ratio; CI, confidence interval; NA, not applicable, indicating variables are not included the regression model.

### 3.4 Multivariate analysis of pathologic risk factors

To clarify whether hypertension, hyperglycemia, and obesity were independent risk factors for parametrial invasion, stromal invasion, and lymph node metastasis, we performed a multivariate logistic regression analysis (see **Table 4**). The results indicated that hypertension was an independent risk factor for parametrial invasion (OR=6.54, 95% CI: 1.60-26.69), whereas hyperglycemia was an independent risk factor for stromal invasion (OR=2.05, 95% CI: 1.07-3.95). Obesity was associated with a low risk of lymph node metastasis (OR=0.07, 95% CI: 0.01-0.60). The presence of obesity in surgical patients can pose difficulties, potentially resulting in inadequate lymph node clearance and failure to detect lymph node metastases (31, 32). To exclude the influence of this variable, we compared the number of lymph node dissections performed on obese and nonobese patients and found no significant difference (median 32 (range 8-89) vs. median 31 (range 14-75); Mann−Whitney U test, *P*=0.654).

**Table 4:** Multivariate regression analysis of risk factors for parametrial invasion, stromal invasion, and lymph node metastasis.

Variable	Parametrial invasion			Stromal invasion			Lymph node metastasis		
	OR	95% CI	*P* value	OR	95% CI	*P* value	OR	95% CI	*P* value
Menopause (yes vs. no)	NA	NA	NA	1.94	1.00-3.74	0.050	NA	NA	NA
Tumor size ≥ 2cm (yes vs. no)	1.83	0.35-9.53	0.474	3.30	1.45-7.48	0.004	3.97	1.12-14.08	0.033
LVSI (yes vs. no)	7.13	1.99-25.56	0.003	2.68	1.38-5.20	0.004	1.87	0.78-4.50	0.160
Parametrial invasion (yes vs. no)	NA	NA	NA	5.87	1.85-18.59	0.003	6.23	1.78-21.81	0.004
Stromal invasion (yes vs. no)	6.57	1.97-21.90	0.002	NA	NA	NA	1.05	0.41-2.66	0.921
Lymph node metastasis (yes vs. no)	3.73	1.17-11.96	0.027	1.13	0.45-2.88	0.794	NA	NA	NA
Hypertension (yes vs. no)	6.54	1.60-26.69	0.009	NA	NA	NA	NA	NA	NA
Hyperglycemia (yes vs. no)	NA	NA	NA	2.05	1.07-3.95	0.032	NA	NA	NA
Obesity (yes vs. no)	NA	NA	NA	NA	NA	NA	0.07	0.01-0.60	0.015

LVSI, Lymphovascular space involvement; OR, odds ratio; CI, confidence interval; NA, not applicable, indicating variables are not included the regression model.

### 3.5 Association between metabolic abnormalities and patient survival

**Figure 1:**
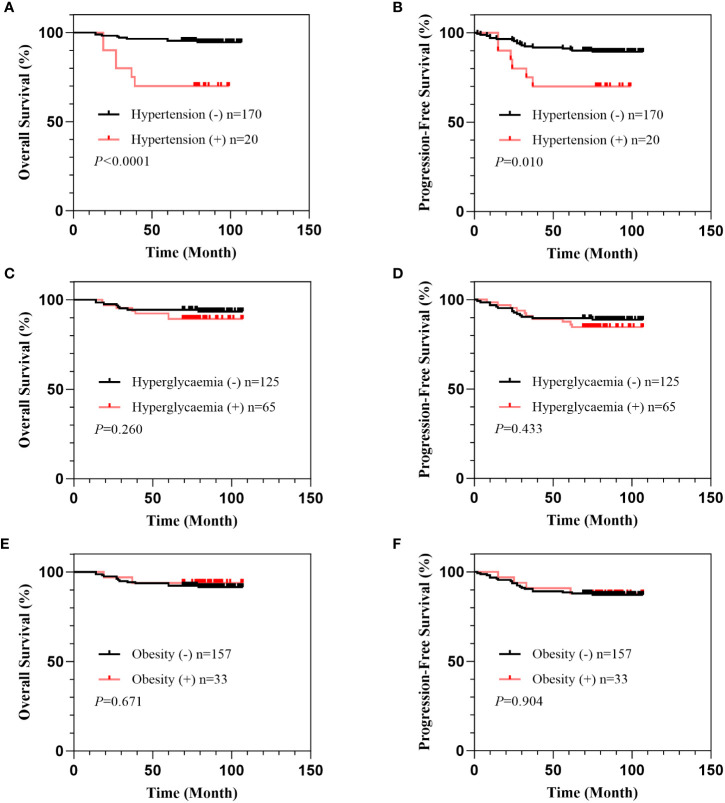
Correlation of hypertension, hyperglycemia, and obesity with survival in patients with IB1 cervical cancer. Overall survival curves of patients stratified by comorbidity of hypertension **(A)**, hyperglycemia **(B)**, and obesity **(C)**. Progression-free survival curves of patients stratified by comorbidity of hypertension **(D)**, hyperglycemia **(E)**, and obesity **(F)**.

To assess the association of hypertension, hyperglycemia, and obesity with the prognosis of early-stage cervical cancer, we performed a survival analysis. With a median follow-up time of 83 months (range, 14-107), we found a 5-year OS of 92.6% and a 5-year PFS of 88.9% in 190 patients with available follow-up data. The patients with hypertension showed had a significantly lower 5-year OS rate (70.0% vs. 95.3%, *P*<0.0001) and a significantly lower 5-year PFS rate than those without hypertension (70.0% vs. 91.2%, *P*= 0.010). Neither hyperglycemia nor obesity was significantly correlated with OS or PFS in the patients with IB1 cervical cancer (**Figure 1**).

## 4 Discussion

In various tumors, a correlation between metabolic syndrome and pathological risk factors was discovered. According to retrospective studies, endometrial cancer patients with metabolic syndrome have higher lymph node metastasis rates, more LVSI, and greater parametrial invasion (33). Another small retrospective study revealed that thyroid cancer patients with metabolic syndrome had larger tumors, a greater proportion of lymph node metastases, and more advanced tumor staging (34). Additionally, for colorectal cancer, the proportion of lymph node metastasis and LVSI were higher in male patients with metabolic syndrome, whereas there was little association in female patients (35). To investigate the relationship between metabolic syndrome and the pathological characteristics of cervical cancer, we retrospectively analyzed the clinicopathological data of clinical early-stage cervical cancer patients. Hypertension was identified as a distinct risk factor for parametrial invasion, while hyperglycemia was determined to be an independent risk factor for stromal invasion. This suggests that abnormalities in body metabolism may play an important role in cervical cancer progression; however, further research is necessary.

We found that patients with hypertension had an increased risk of parametrial invasion and poorer overall survival. According to epidemiological studies, hypertension is associated with the occurrence of malignant tumors at various sites and can affect the prognosis of these cancers, particularly kidney, colon, esophageal, breast, skin, prostate, liver, uterine, and pancreatic cancers (36, 37). Furthermore, regarding the connection between hypertension and pathological characteristics, a retrospective study revealed that hypertension was associated with advanced TNM staging in gastric cancer (38). Likewise, large retrospective studies have discovered that hypertension is linked to a higher risk of larger tumor size, positive lymph node metastasis, and later tumor stage in thyroid cancer (39). The potential mechanistic correlation between hypertension and tumor progression may involve the influence of hormones, such as angiotensin II, which can enhance tumor angiogenesis by stimulating the production of vascular endothelial growth factor (40, 41). Additionally, estrogen, known for its protective effects against hypertension, may play a role in regulating the proliferation and apoptosis of tumor cells through receptor binding (42, 43). Additionally, hypertension-induced chronic inflammation, the upregulation of hypoxia-inducible factors, and reactive oxygen species promote tumor progression (44). In addition, cancer patients are more likely to develop hypertension because anti-VEGF drugs and targeted medications can dramatically increase blood pressure (45, 46). Meanwhile, the drugs used in hypertensive patients may affect tumor progression (47). As a result, hypertension may contribute to cancer progression through multiple mechanisms.

Not only is hypertension linked to cervical cancer invasion, but we discovered that hyperglycemia increased the risk of stromal invasion. Epidemiological studies revealed a correlation between diabetes and decreased overall survival as well as an increased susceptibility to cancer-related mortality, including pancreatic, ovarian, breast, and uterine cancers (48–50). Moreover, the link between hyperglycemia and tumor pathology risk factors has been assessed in several tumors. In a large retrospective study on breast cancer, Hou et al. found that diabetic patients had an advanced tumor stage, a higher incidence of lymph node metastases, and a poor prognosis overall (51, 52). Furthermore, a retrospective study found a significant association between diabetes mellitus and an increased risk of stromal invasion in colorectal cancer (53), which is consistent with our findings. The mechanism by which hyperglycemia promotes tumor progression may be related to insulin resistance. Insulin and IGF-1 receptors can be observed in most types of cancer tissue. Insulin receptors in cancer cells are engaged to activate proliferative signaling pathways, protect against apoptotic stimuli, and promote cancer invasion and metastasis (54). Moreover, hyperglycemia induces the production of numerous inflammatory factors, such as IL-6 and TNF-α, that can induce epithelial mesenchymal transition, thereby promoting tumor cell invasion and inhibiting apoptosis (55). Additionally, hyperglycemia induces cellular hypoxia, and the exacerbation of microenvironmental hypoxia leads to an increase in HIF-1 expression, which promotes pancreatic cancer metastasis (56). In addition, high blood glucose can provide nutrients for the rapid proliferation of malignant tumor cells, thereby accelerating the proliferation and invasion of tumor cells (57).

Obesity is an epidemiologic risk factor for the development of many cancers, and it has a negative impact on the prognosis of many, but not all, cancers (58–60). We noted that obesity correlated with a low risk of lymph node metastasis in obese and nonobese patients, with no significant difference in the number of lymph nodes removed. This finding is consistent with the results of a previous retrospective study on gastric cancer, which also found obesity to be a factor protecting against lymph node metastasis (61). However, this is contrary to the findings of Lucas et al., who discovered that higher BMI was an independent risk factor for lymph node metastasis in stage IA2 or IB1 cervical cancer (62). Mechanistically, the correlation between organismal metabolism and lymph node metastasis is unclear. Experimental studies have revealed that the monounsaturated fatty acid (MUFA) oleic acid in lymphatic vessels mitigates oxidative stress and promotes cancer cell circulation in lymphatic systems (63). Inmaculada et al. hypothesized that a high-fat diet rich in MUFAs may prevent ferroptosis to potentially promote tumor growth and metastasis, but this has not been demonstrated (64). Currently, the relationship between obesity and lymph node metastasis remains unclear. Therefore, large prospective studies and fundamental research are required for further investigation.

Additionally, there are limitations to this study. First, this is a single-center study with a sample size of 246 patients, which, by its nature, cannot avoid selective bias or confirm causality; therefore, a multi-center investigation employing a more substantial sample size is required to validate the results. Second, the association between metabolic syndrome and pathological characteristics was not investigated due to a lack of information on lipid metabolism and waist circumference in medical records. Although the overall analysis of metabolic disorders did not reveal a significant correlation with each pathological feature, we discovered that hypertension, hyperglycemia, and obesity were all associated with cancer invasion. This suggests that any metabolic components of the metabolic syndrome should be analyzed apart from the syndrome itself.

## 5 Conclusion

Hypertension and hyperglycemia may increase the risk of parametrial invasion or stromal invasion in patients with early clinical stage cervical cancer, indicating that metabolic abnormalities may play an important role in the local progression of early cervical cancer; however, multi-center, large scale studies are needed to verify the observation.

## Data availability statement

The raw data supporting the conclusions of this article will be made available by the authors, without undue reservation.

## Ethics statement

The studies involving humans were approved by The Ethics Committee of Tongji Medical College, Huazhong University of Science and Technology. The studies were conducted in accordance with the local legislation and institutional requirements. Written informed consent for participation was not required from the participants or the participants’ legal guardians/next of kin in accordance with the national legislation and institutional requirements.

## Author contributions

TS: Writing – original draft, Writing – review & editing, Data curation, Formal Analysis, Investigation, Methodology. JZ: Supervision, Writing – review & editing, Investigation, Methodology. WL: Investigation, Writing – review & editing, Validation. XW: Validation, Writing – review & editing. YG: Validation, Writing – review & editing. ZW: Conceptualization, Project administration, Writing – review & editing. SH: Project administration, Supervision, Writing – review & editing. JC: Conceptualization, Funding acquisition, Project administration, Resources, Supervision, Writing – original draft, Writing – review & editing.
